# Research on Trajectory Recognition and Control Technology of Real-Time Tracking Welding

**DOI:** 10.3390/s22218546

**Published:** 2022-11-06

**Authors:** Xiaohui Zhao, Yaowen Zhang, Hao Wang, Yu Liu, Bao Zhang, Shaoyang Hu

**Affiliations:** 1 Key Laboratory of Automobile Materials, School of Materials Science and Engineering, Jilin University, Changchun 130025, China; 2 School of Mechanical and Aerospace Engineering, Jilin University, Changchun 130025, China; 3 Key Laboratory of Solidification Control and Digital Preparation Technology (Liaoning Province), School of Materials Science and Engineering, Dalian University of Technology, Dalian 116024, China

**Keywords:** structured light vision, adaptive feature extraction, embedded Pauta criterion, robotic welding

## Abstract

Real-time tracking welding with the assistance of structured light vision enhances the intelligence of robotic welding, which significantly shortens teaching time and guarantees accuracy for user-customized product welding. However, the robustness of most image processing algorithms is deficient during welding practice, and the security regime for tracking welding is not considered in most trajectory recognition and control algorithms. For these two problems, an adaptive feature extraction algorithm was proposed, which can accurately extract the seam center from the continuous, discontinuous or fluctuating laser stripes identified and located by the CNN model, while the prior model can quickly remove a large amount of noise and interference except the stripes, greatly improving the extraction accuracy and processing speed of the algorithm. Additionally, the embedded Pauta criterion was used to segmentally process the center point data stream and to cyclically eliminate outliers and further ensure the accuracy of the welding reference point. Experimental results showed that under the guarantee of the above-mentioned seam center point extraction and correction algorithms, the tracking average error was 0.1 mm, and even if abnormal trajectory points existed, they did not cause welding torch shaking, system interruption or other accidents.

## 1. Introduction

The recognition and control of welding trajectory is a major problem in intelligent welding, and structured light vision is an efficient way to deal with it. With the assistance of structured light vision sensors, teaching-playback robots are capable of intelligently realizing the automatic planning of welding trajectory by laser multi-point positioning [1,2], pre-welding trajectory fitting [3] and real-time tracking [4], of which real-time tracking is the most important technical means. A highly robust image processing algorithm for feature point extraction is a technical prerequisite for the tracking system, but it brings reservations in time-consuming practical welding, where the tracking reference points are not extracted 100% correctly due to external dynamic interference and the number of outliers increases with the welding time. Therefore, a stable image processing algorithm, as well as a flexible outlier tracking point removal algorithm, must be incorporated into the tracking regime at the same time.

The robot’s motion control model based on coordinate points is relatively robust and mature, but the extraction of the seam center would be strongly disturbed by arc, splash, light, etc. The extraction of the center point is mainly divided into two steps: automatically locating the laser stripe region of interest (ROI) and detecting feature points from ROI. In order to be able to shorten the image processing cycle and improve the accuracy of feature point extraction, a target tracking algorithm is used to acquire ROI. There are two main forms of regions of interest: large pixel regions containing joint information with high adaptability and flexibility and tiny pixel regions containing single feature points with high specificity and personalization. For thick plates with large bevels, the strong arc splash across the bevel gap causes violent reflected light at the bevel inflection point, and the algorithm aiming at tracking the node (bevel inflection point) has a certain feature point extraction error, generally within 10 pixels, but the actual error can be up to 1 mm (the magnification factor in the camera vision middle area is 1 pixel ≈ 0.1 mm). Therefore, the algorithm that tracks the bevel was used in this study to further reduce the error of feature point extraction by the complete laser stripe curve. A weighted local cosine similarity algorithm was adopted to detect seam laser area [5]. Zou et al. [6] used the spatiotemporal context (STC) object tracking algorithm to obtain the processing object of feature point extraction. The recurrent neural network (RNN) was used to learn the temporal context information of convolutional features to accurately detect the seam region [7]. Zou et al. [8] proposed a continuous convolution operator tracker (CCOT) object-tracking algorithm to get the object of image processing. Li et al. [9] searched the profile of the welding seam in a small area by using a Kalman filter. The location of welding points in the presence of large distractions from ambient illumination, metal dust, splash and strong arc needs to be rapidly and accurately determined to improve the robustness of laser stripe centerline extraction and seam center extraction algorithms. Appropriate target-tracking algorithms can significantly improve the robustness of the welding system with auxiliary light sources. Some target detection algorithms, such as KCF [10], SVM [11], and ERFNet [12], have complicated training processes; therefore, a real-time object detection algorithm based on CNN [13] was used in this research to rapidly and accurately determine the region of interest, including bevel laser stripes.

**Figure 1:**
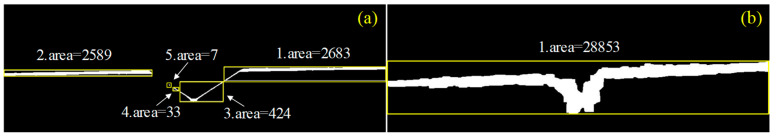
Connected region detection results of V-shaped joint. (**a**) Connected area extraction results for the narrow laser line. (**b**) Connected area extraction results for the wide laser line.

**Figure 2:**
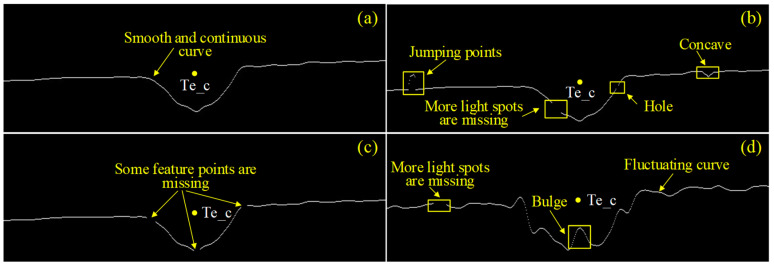
Four typical types of laser stripes under practical operating conditions. (**a**) The theoretical laser stripe. (**b**) The laser stripe with jumping points. (**c**) The laser stripe with missing feature points. (**d**) The fluctuating laser stripe.

These ROI tracking and prediction algorithms are highly robust, but many of the algorithms for detecting feature points from the joint region need to be optimized to meet the requirements of actual welding conditions. Zhang et al. [14,15] detected the laser stripe based on the connected region theory, but the largest connected region did not always represent the laser stripe during welding (as outlined in Figure 1), and the theory easily removed important information. Wu et al. [16] used the Hough linear detection method to obtain the laser stripe centerline equation. Jawad et al. [17] improved the Otus and line detection algorithms to extract feature points. A precise Hough transform algorithm was designed by Fan et al. [18] to extract the seam center. Furthermore, Xiao et al. [19] extracted the seam center based on the inflection point theory after detecting the laser stripe by the Steger algorithm. However, the feature extraction algorithm based on the theoretical laser stripe model (Figure 2a) was prone to failure when there were holes, jumping points, missing feature points and other defects in the laser stripe, as shown in Figure 2b–d. For these reasons, an adaptive feature extraction algorithm was investigated in order to extract the seam center from the bevel laser stripe with various defect characteristics under actual welding.

Although seam area prediction models and seam center extraction algorithms proposed by Zhang et al. [20,21,22] could extract the motion reference point to a large extent, the accuracy of these models and algorithms was not 100%. Due to the irresistible, dynamic, irregular and uncertain interference in the acquisition of accurate feature points, there was a high probability that the original laser image lacked valid information, and thus the authentic seam feature points were not obtained, which would lead to the chaos of motion program and was why the technology suddenly failed during the welding process. Factors such as strong arc spatter, sensor contamination and ambient light could cause the target tracking model and feature point extraction algorithm to extract the wrong seam center point, so the center point data stream obtained through image processing should not be directly transmitted to the tracking welding controller, for which the security or robustness guarantee mechanism for analyzing the authenticity and accuracy of feature points needs to be put forward.

Taking the V-shaped seam as the research object, it was proposed to use the CNN model to automatically locate the ROI of laser stripes. An adaptive feature extraction algorithm based on row and column scanning extracted the seam center point. The prior model further improved the extraction accuracy of feature points at the pixel level. Subsequently, the embedded Pauta criterion [23] processed the center point data stream to ensure the authenticity and accuracy of tracking reference points by eliminating outliers. Finally, the adaptability of these algorithms was discussed. Experiments were conducted in a real-time tracking welding system to verify the robustness and accuracy of the algorithms.

## 2. Structured Light Vision Welding System

**Figure 3:**
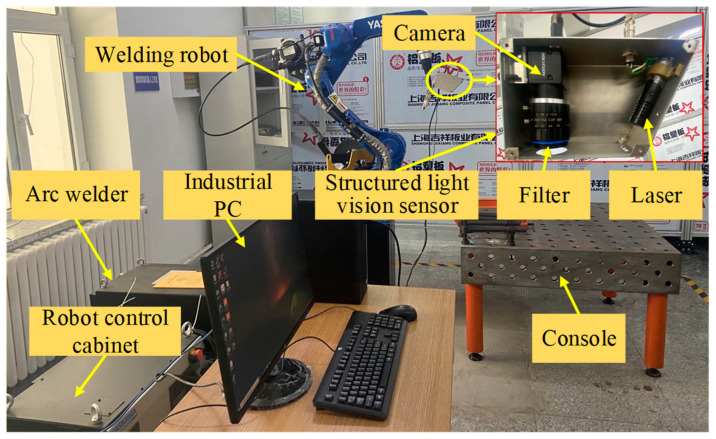
Diagram of a structured light vision welding system.

A structured light vision welding system was independently developed, which consisted of a six-DOF welding robot (Yaskawa, AR1440, Liaoning Dazheng Intelligent Robot Co. Ltd., Liaoning, China), a structured light vision sensor [24], a robot control cabinet (YRC1000, Liaoning Dazheng Intelligent Robot Co. Ltd., Liaoning, China), an arc welder (RD350S, Liaoning Dazheng Intelligent Robot Co. Ltd., Liaoning, China), an industrial PC and a console, as shown in Figure 3. The structured light vision sensor was based on the coupling of the camera (MV-CA050-20UM, Hangzhou Hikvision Digital Technology Co., Ltd., Hangzhou, China), an infrared laser (HD650AB100-16GD-WLD, Shenzhen Infrared Laser Technology Co., Ltd., Shenzhen, China) and a bandpass filter (FU-650LGP-Y34, Shenzhen Infrared Laser Technology Co., Ltd., Shenzhen, China) attached to the camera lens. The camera and the torch are coaxial, and the laser forms an angle of 25° with the axis of the camera. The intersection of the laser’s optical axis and the camera’s axis was positioned at 170.33 mm from the camera lens. In addition, the look-ahead distance between the laser line and welding arc was 50.5 mm when real-time tracking welding.

**Figure 4:**
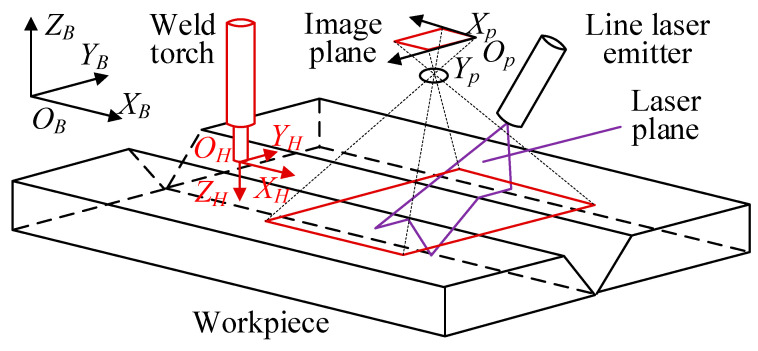
Schematic of robot automatic welding.

Figure 4 is a schematic of robot automatic welding, which illustrates the relationship between the pixel coordinate system *O_P_X_P_Y_P_*, the tool coordinate system *O_H_X_H_Y_H_Z_H_* and the robot base coordinate system *O_B_X_B_Y_B_Z_B_*. On the basis of the structured light perspective projection imaging model, the image coordinate system *O_I_X_I_Y_I_* existed on the *O_P_X_P_Y_P_* plane, and the coordinate origin was located at the pixel center; in addition, the camera coordinate system *O_C_X_C_Y_C_Z_C_* was located at a distance *f* (focal length) directly above the image coordinate system. When *P* was assumed as a point on this laser line, then its coordinates in the *O_I_X_I_Y_I_* coordinate system, *O_P_X_P_Y_P_* coordinate system and *O_C_X_C_Y_C_Z_C_* coordinate system were (*u*, *v*), (*c*, *r*) and (*x_c_, y_c_, z_c_*), respectively.

The transformation between (*x_c_, y_c_, z_c_*) and (*u*, *v*) was established via the trigonometry principle as follows:

(1) $$\left[\begin{array}{c} u\\ v\\ 1 \end{array}\right]=\frac{1}{z_{c}}\left[\begin{array}{c} f\\ 0\\ 0 \end{array}\;\begin{array}{c} 0\\ f\\ 0 \end{array}\;\begin{array}{c} 0\\ 0\\ 1 \end{array}\;\begin{array}{c} 0\\ 0\\ 0 \end{array}\right]\left[\begin{array}{c} x_{C}\\ y_{C}\\ z_{C}\\ 1 \end{array}\right]$$

Under non-ideal conditions, the real coordinates (*u_d_*, *v_d_*) of the *P* point in the image coordinate system *O_I_X_I_Y_I_* were geometrically distorted from the theoretical coordinates (*u*, *v*) [25], and they were related as follows:

(2) $$\left[\begin{array}{c} u_{d}\\ v_{d} \end{array}\right]=\frac{2}{1+\sqrt{1-4k\left(u^{2}+v^{2}\right)}}\left[\begin{array}{c} u\\ v \end{array}\right]$$

where *k* is the distortion coefficient.

For obtaining the coordinates of the *P* point, the camera coordinate system *O_C_X_C_Y_C_Z_C_*, the transformation between the pixel coordinate system *O_P_X_P_Y_P_* and the image coordinate system *O_I_X_I_Y_I_* needed to be established as follows [26]:

(3) $$\left[\begin{array}{c} c\\ r\\ 1 \end{array}\right]=\left[\begin{array}{ccc} 1/S_{x} & 0 & u_{0}\\ 0 & 1/S_{y} & v_{0}\\ 0 & 0 & 1 \end{array}\right]\left[\begin{array}{c} u_{d}\\ v_{d}\\ 1 \end{array}\right]$$

where *S_x_*, and *S_y_* represent the imaging magnification coefficients in the horizontal and vertical directions, respectively. (*u*_0_, *v*_0_) represents the pixel coordinates of the intersection of the optical axis and photosensitive chip.

To determine the exact mapping relationship between 2D pixel coordinates (*c*, *r*) and 3D camera coordinates (*x_c_, y_c_, z_c_*), a constraint equation needed to be established by light plane calibration, as follows:

(4) $$A*x_{C}+B*y_{C}+C*z_{C}-D=0$$

The transformation between (*x_c_, y_c_, z_c_*) and (*c*, *r*) can be derived using Equations (1)–(4):

(5) $$\{\begin{array}{c} x_{C}=\frac{D\left(S_{x}c-u_{0}S_{x}\right)}{A\left(S_{x}c-u_{0}S_{x}\right)+B\left(S_{y}r-v_{0}S_{y}\right)+Cf\delta }\\ y_{C}=\frac{D\left(k_{y}r-v_{0}k_{y}\right)}{A\left(S_{x}c-u_{0}S_{x}\right)+B\left(S_{y}c-v_{0}S_{y}\right)+Cf\delta }\\ z_{C}=\frac{Df}{A\left(S_{x}c-u_{0}S_{x}\right)+B\left(S_{y}r-v_{0}S_{y}\right)+Cf\delta } \end{array}$$

where $\delta =ku_{0}^{2}S_{x}^{2}-2ku_{0}S_{x}^{2}c+kv_{0}^{2}S_{y}^{2}-2kv_{0}S_{y}^{2}r+kS_{x}^{2}c^{2}+kS_{y}^{2}r^{2}+1$, (*S_x_*, *S_y_*, *k*, *u*_0_, *v*_0_) are collectively referred to as camera internal parameters, which were determined by the HALCON machine vision library calibration and (*A*, *B*, *C*, *D*) are laser line parameters, which were obtained by optical plane calibration.

The mapping relationship between the coordinates of the *P* point in the world coordinate system *O_B_X_B_Y_B_Z_B_* and the coordinates in the camera coordinate system *O_C_X_C_Y_C_Z_C_* is shown in Equation (6).

(6) $$\left[\begin{array}{c} x_{B}\\ y_{B}\\ z_{B}\\ 1 \end{array}\right]=\left[\begin{array}{c} R\;\;T\\ 0\;\;1 \end{array}\right]X_{S}\left[\begin{array}{c} x_{C}\\ y_{C}\\ z_{C}\\ 1 \end{array}\right]$$

where *X_S_* is the hand-eye transformation matrix (i.e., the transformation matrix of the camera coordinate system *O_C_X_C_Y_C_Z_C_* to the tool coordinate system *O_H_X_H_Y_H_Z_H_*) obtained from the hand-eye calibration. The expressions of *R* and *T* are as follows:

(7) $$R=R_{3}\cdot R_{2}\cdot R_{1}$$

(8) $$T={\left[X\;Y\;Z\right]}^{T}$$

(9) $$R_{1}=\left[\begin{array}{c} 1\\ 0\\ 0 \end{array}\;\begin{array}{c} 0\\ \cos R_{X}\\ \sin R_{X} \end{array}\;\begin{array}{c} 0\\ -\sin R_{X}\\ \cos R_{X} \end{array}\right]$$

(10) $$R_{2}=\left[\begin{array}{c} \cos R_{Y}\\ 0\\ -\sin R_{Y} \end{array}\;\begin{array}{c} 0\\ 1\\ 0 \end{array}\;\begin{array}{c} \sin R_{Y}\\ 0\\ \cos R_{Y} \end{array}\right]$$

(11) $$R_{3}=\left[\begin{array}{c} \cos R_{Z}\\ \sin R_{Z}\\ 0 \end{array}\;\begin{array}{c} -\sin R_{Z}\\ \cos R_{Z}\\ 0 \end{array}\;\begin{array}{c} 0\\ 0\\ 1 \end{array}\right]$$

where (*X*, *Y*, *Z*, *R_X_*, *R_Y_*, *R_Z_*) are the six parameters of the robot’s motion in the Cartesian coordinate system, which were read out by the trainer.

The pixel coordinates (*c*, *r*) of the feature points obtained from image processing and the real-time robot motion parameters (*X*, *Y*, *Z*, *R_X_*, *R_Y_*, *R_Z_*) were brought into Equations (5) and (6) to obtain the dynamic 3D coordinates of the seam feature points in the robot base coordinate system.

Classical deviation control structures (e.g., PID, Fuzzy, Fuzzy-PID) could also be used to adjust the welding gun to the correct welding position after obtaining the real-time deviation of the welding gun through the image processing algorithm, but the traditional deviation control structures were more complex and suitable for lower welding speeds. For this reason, this research utilized the structured light vision sensor to scan the joint, obtained the three-dimensional coordinate data stream of center points in real-time during welding, and enabled the gun to track these actual welding points; this tracking welding control [27] method was not only simple in structure, but also had a fast response time.

**Figure 5:**
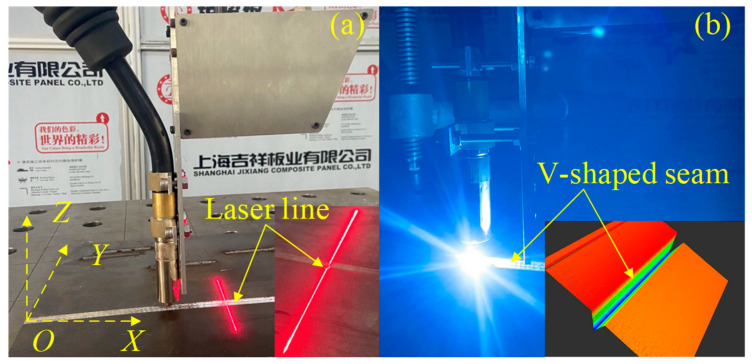
Butt joint workpiece with a V-shaped seam. (**a**) The butt joint workpiece. (**b**) The 3D point cloud data of V-shaped seam.

Figure 5 shows the butt joint workpiece with a V-shaped seam. The 3D point cloud data in *O_B_X_B_Y_B_Z_B_* can be obtained with Equations (5) and (6) by scanning the groove, as shown in Figure 5b.

## 3. Tracking and Identification of the Seam Area

**Figure 6:**
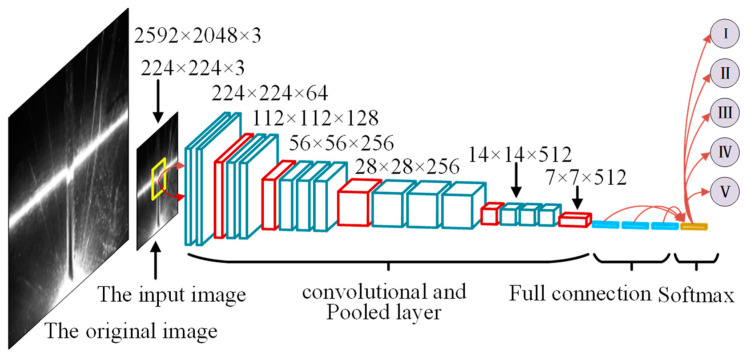
Schematic diagram of the CNN model.

The CNN model for the typical V-shaped seam was established, as outlined in Figure 6. In order to improve the accuracy of locating the ROI from a strong noise image, this paper adopted the following flow:

1. Resized the original image to 224 × 224 from 2592 × 2048;
2. Multiple convolutions based on the kernel of size 3 × 3, after that, the output of the picture was changed to a one-dimensional vector;
3. Softmax layer completed the identification and localization of the target area through the processing of the full connection.

**Figure 7:**
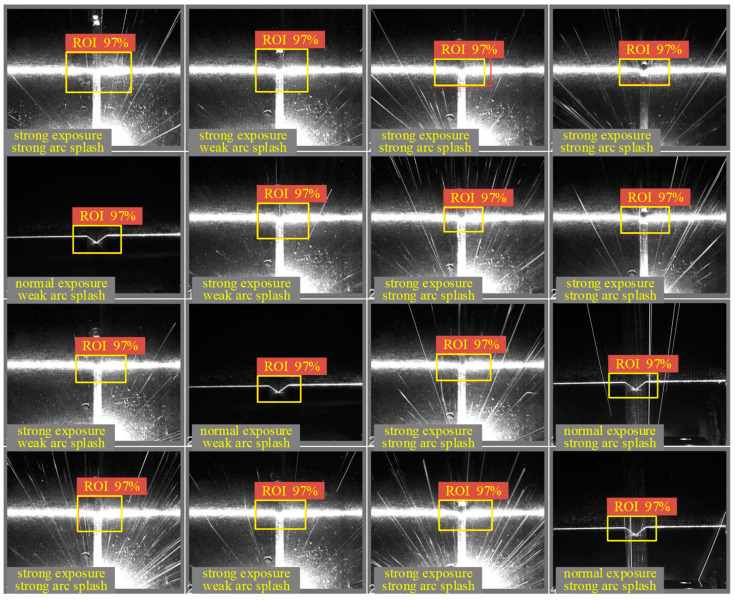
Target area identifying results.

The sample set for the training in the study consisted of 4134 laser images with varying degrees of interference. The ROI object tracking test on 472 images from actual welding showed that the predictive validation accuracy of the model was about 97.0%, as presented in Figure 7. From laser stripe images, it was seen that metal soot and a large amount of splatter were present in the joint bevel area, especially at the corners and bottom of the joint, where there was a large amount of reflected light. It is worth noting that the light intensity of some splatters was even greater than that of the laser streak itself, and when they swept across the bottom of the bevel and the edges on both sides, the target tracking algorithm that directly acquires bevel feature points by locating tiny pixel areas was likely to extract target points with large errors.

## 4. Laser Stripe Feature Points Extraction

**Figure 8:**
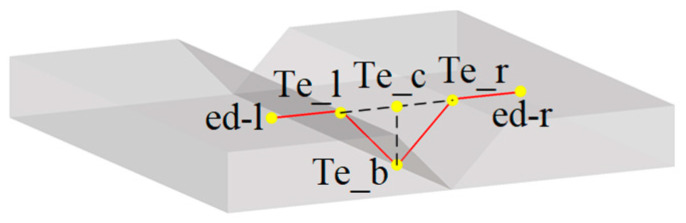
The feature points of the V-shaped seam.

The points ed-l, ed-r, Te_l, Te_r, Te_b and Te_c were specified as the feature points of the V-shaped seam, among which Te_c was the seam center point as the reference point for the welding trajectory, as shown in Figure 8.

### 4.1. Laser Stripe Centerline Extraction

The gray barycentric algorithm [28] was utilized to quickly and efficiently extract the stripe centerline. The “centroid” in the pixel area was considered the pixel center in the area, and the calculation formula of the area centroid is expressed as follows:

(12) $$\{\begin{array}{c} \bar{c}=\frac{\sum_{\left(c,r\right)\in \Omega }c\cdot f\left(c,r\right)}{\sum_{\left(c,r\right)\in \Omega }f\left(c,r\right)}\\ \bar{r}=\frac{\sum_{\left(c,r\right)\in \Omega }r\cdot f\left(c,r\right)}{\sum_{\left(c,r\right)\in \Omega }f\left(c,r\right)} \end{array}$$

where f(c,r) represents the gray value of the pixel point with coordinates (*c*, *r*), *Ω* denotes the set of target regions, and ($\bar{c}$, $\bar{r}$) is the gray barycentric coordinate of the region.

**Figure 9:**
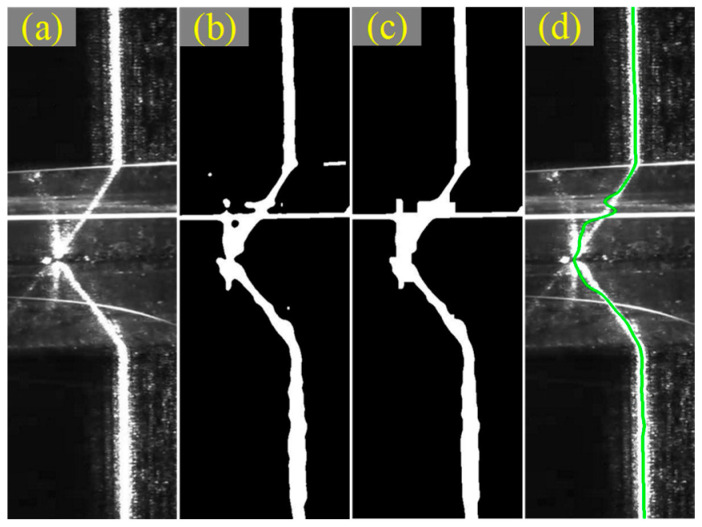
The extraction process of stripe centerline. (**a**) Initial image. (**b**) Otsu threshold segmentation after Median filtering. (**c**) Morphological processing. (**d**) Extracting stripe centerline.

The skeleton of the laser stripes obtained by the algorithm scanning along the *c*-direction is equal to the set of the grayscale center of gravity points in each column region of the image. For the laser stripe image covered by a strong arc splash, the interference should be removed by the image preprocessing algorithm before the skeleton of the stripe was extracted by the gray barycentric method, as illustrated in Figure 9. It is seen in Figure 9d that the extracted centerline in the search area could accurately map the spatial coordinate information of the seam. Furthermore, with the column of the pixel as an independent variable and the row of the pixel as a dependent variable, there was a one-to-one mapping functional relationship between the independent variable and the dependent variable for the skeleton.

### 4.2. Seam Center Point Recognition

**Figure 10:**
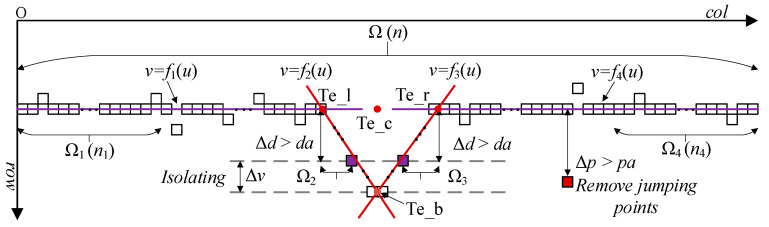
The design idea of adaptive feature extraction algorithm.

When the laser skeleton possessed discontinuous characteristics such as jumping points, holes and missing points (prone to occur in weak arc splash welding, as shown in Figure 2b,c) or fluctuating characteristics (prone to occur in strong arc splash welding, as shown in Figure 2d), traditional feature point extraction methods, such as inflection point method, slope analysis method and Hough straight line detection method, were likely to fail. In this study, an adaptive feature extraction algorithm based on row scanning and column scanning was adopted to solve this problem. The design idea of the adaptive feature extraction algorithm is shown in Figure 10.

**Figure 11:**
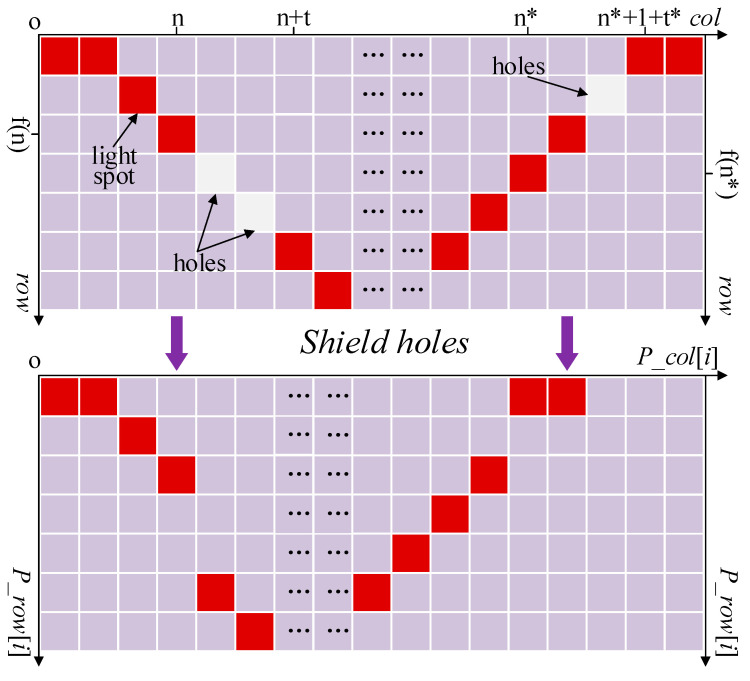
The process of shielding holes.

The steps involved in the algorithm are as follows:

Step 1: Shield holes on the skeleton (as illustrated in Figure 11). After that, the horizontal coordinate set p_col and vertical coordinate set p_row to be processed can be obtained.

Step 2: Remove the jumping points. If the point (p_col[a], p_row[a]) satisfies Equation (13), this point is treated as a jumping point and removed; otherwise, this point will be used as the comparison point of the next point to be determined. Through Equation (13), the new abscissa set p_c and ordinate collections p_r are obtained.

(13) $$\begin{array}{c} \Delta p=\left|p\_row\left[a\right]-p\_row\left[b\right]\right|\\ \Delta p>\left|\left(p\_col\left[a\right]-p\_col\left[b\right]\right)*k_{\alpha /2}*\vartheta \right|=pa \end{array}$$

where $k_{\alpha /2}$ and ϑ are the slope of the groove surface and correction factor, respectively; (p_col[b], p_row[b]) is the nearest normal point in front of (p_col[a], p_row[a]); a and b are integers, and a > b.

Step 3: Perform the least-squares fitting method on the first *n*_1_ points and the last *n*_4_ points in Ω respectively, and obtain the straight-line equations $v=f_{1}\left(u\right)$ and $v=f_{4}\left(u\right)$.

Step 4: Utilizing row scanning, Δv rows of data at the bottom of the skeleton are isolated to eliminate the influence of reflected light in the valley.

Step 5: Scanning from the point ($p\_col\left[n_{1}\right]$, $p\_row\left[n_{1}\right]$) to the valley bottom and from the valley bottom to the point ($p\_col\left[n-n_{4}\right]$, $p\_row\left[n-n_{4}\right]$), the least-squares fitting is performed on the points satisfying Equation (14) to obtain equations $v=f_{2}\left(u\right)$ and $v=f_{3}\left(u\right)$.

(14) $$\frac{\left|a*p\_c\left[s\right]-p\_r\left[s\right]+b\right|}{\sqrt{a^{2}+1}}=\Delta d>da$$

where da is set to 10; When calculating $f_{2}$, *a* and *b* represent the slope and intercept of *f*_1_, respectively, and when calculating $f_{3}$, *a* and *b* represent the slope and intercept of $f_{4}$, respectively.

Step 6: Calculate the coordinate values of Te_c according to Equation (15).

(15) $$\{\begin{array}{c} u_{\mathrm{Te}\_c}=\frac{u_{\mathrm{Te}\_l}+u_{\mathrm{Te}\_r}}{4}+\frac{u_{\mathrm{Te}\_b}}{2}\\ v_{\mathrm{Te}\_c}=\frac{v_{\mathrm{Te}\_l}+v_{\mathrm{Te}\_r}}{2}\;\;\;\;\;\;\;\;\;\;\;\;\;\; \end{array}$$

where Te_l, Te_r and Te_b are obtained by intersecting the lines calculated above.

**Figure 12:**
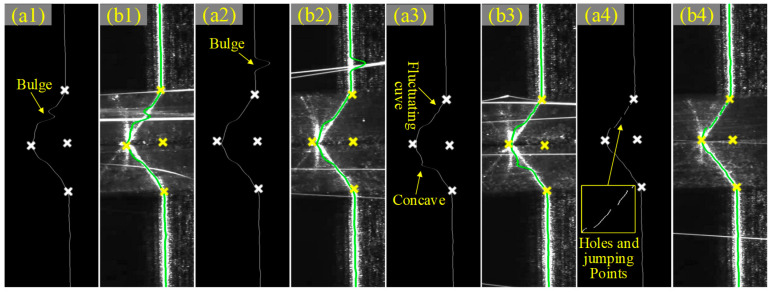
The extraction results of seam feature points. (**a1**–**a4**) Positions of feature points on the centerline. (**b1**–**b4**) Mapping positions of feature points and centerlines in (**a1**–**a4**) on initial images.

The laser image with a strong arc splash was taken as an example. It can be found from Figure 12 that the algorithm above has a high anti-interference ability to arc splash, and the extracted feature point Te_c could truly represent the actual location of the seam center. At the same time, even in the case of holes, bulges, concaves and jumping points in the laser centerline or in the case of fluctuations in the laser centerline, the algorithm also has a good extraction effect.

### 4.3. Improving Te_c Accuracy

Strong arc and strong splash made the laser stripe centerline deviate from the ideal position, but the slope and width of the stripe were almost unchanged in adjacent frames. Thence, the noise of the next frame image was effectively filtered according to the context information.

**Figure 13:**
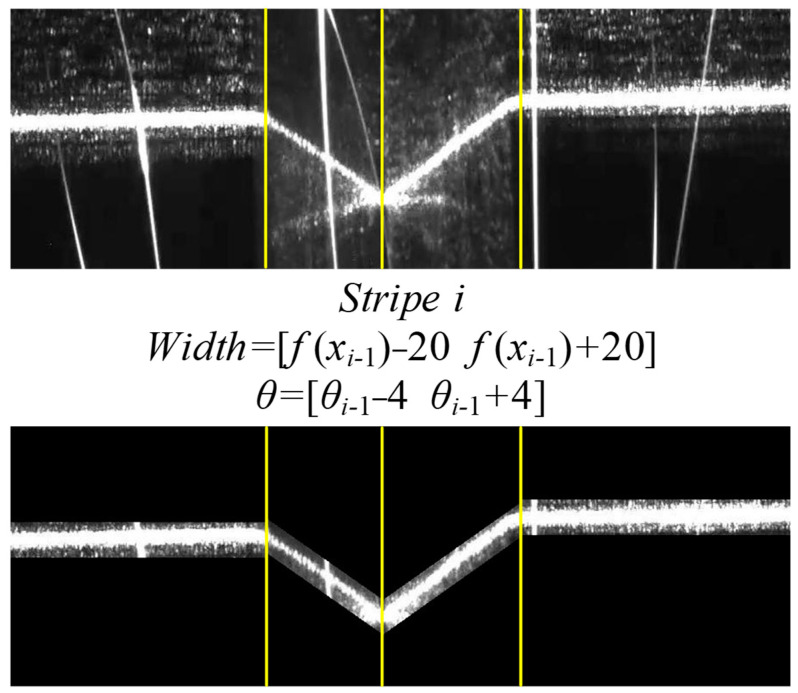
The prior model.

The determination of the slope and width of each stripe was defined in a prior algorithm, as Equation (16). Only the points that satisfied the algorithm were retained. The process of the prior model is illustrated in Figure 13.

(16) $$Stripe\;i:\left\{\begin{array}{c} width=\left[y_{min}\;\;\;\;y_{max}\right]\\ \;\;\;\;\;\theta \;\;\;=\left[\theta_{min}\;\;\;\;\theta_{max}\right] \end{array}\right\}$$

where i is the sequence number of the stripe image; width represents the width range of the stripe. θ represents the angle range of the stripe.

**Figure 14:**
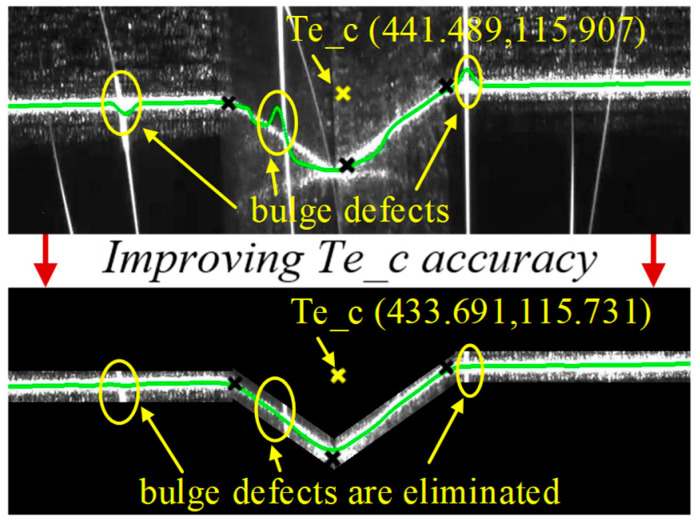
The effect of improving the accuracy of the Te_c.

According to the stripe feature points of the previous frame, the current frame image was divided into four segments. Based on the centerline angle of the connected region in each segment and the stripe centerline equation of the previous frame, the main stripe information was separated from the background noise by the angle criterion and width criterion of the prior algorithm. The effect of improving the accuracy of Te_c (Figure 14) showed that the prior algorithm overcame the strong arc and splash interference, ensured that the stripe centerline was more consistent with the theoretical attitude and position (stripe centerline defect characteristics were removed) and further reduced the error of center point extraction at the pixel level.

In order to improve the stability and accuracy of the feature point extraction algorithm, in this study, the CNN model was used to locate and track the bevel region of interest in real-time, followed by the prior model to remove the image interference information, and finally, the adaptive feature extraction algorithm was able to extract the seam center quickly. Since the above algorithms were discussed under conventional welding conditions, it was necessary to perform tests under extreme environments to explore the adaptability of the algorithms to the welding environment.

**Figure 15:**
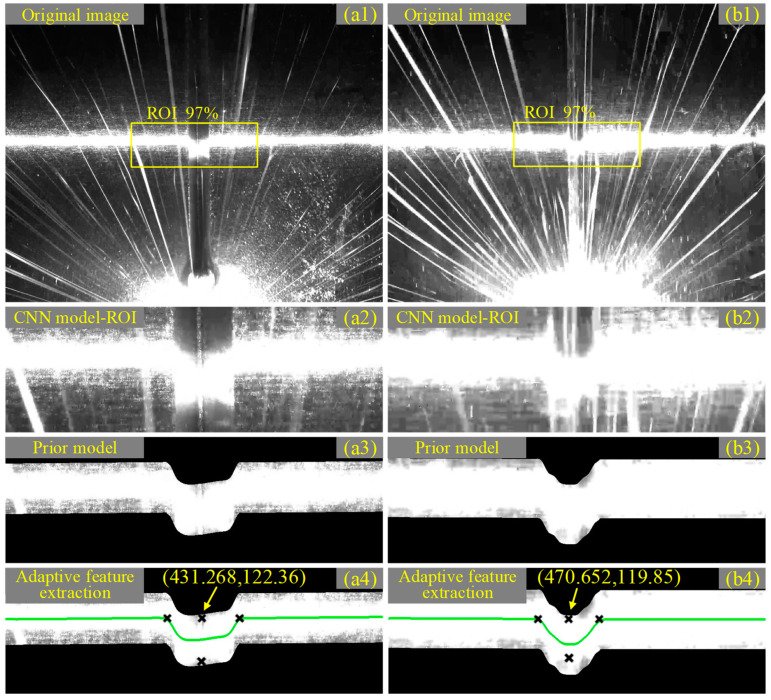
Test results under extreme environments. (**a1**,**b1**) Original image. (**a2**,**b2**) ROI. (**a3**,**b3**) The priori model processing effect. (**a4**,**b4**) The feature point extraction effect.

An extreme welding environment should meet the following two conditions: (1) camera exposure values higher than normal (15,000 EV) and (2) thicker laser line (>1 mm). Under such welding conditions, tests a and b were performed, where in test a, the welding current was 100 A, the camera exposure value was 20,000 EV and the laser line was 1.5 mm; in test b, the welding current was 160 A, the camera exposure value was 40,000 EV and the laser line was 2.0 mm, as shown in Figure 15. As the welding current, exposure value and excitation line width increased, the noise (strong arc splash) in the laser streak image interfered more and more with the bevel laser lines; for example, some laser lines could no longer effectively characterize the bevel morphology (Figure 15(b2)). However, the pose and position of the stripe centerline extracted from the CNN model and prior model are more realistic representations of the 3D shape of the joint bevel (Figure 15(a4,b4)), while the adaptive feature extraction algorithm was able to extract the seam center more accurately based on the curve characteristics of the laser stripe centerline within the ROI. It can be concluded that the seam center extraction scheme proposed above was highly practical and adaptable for various complex welding environments.

## 5. Outliers Filtering

**Figure 16:**
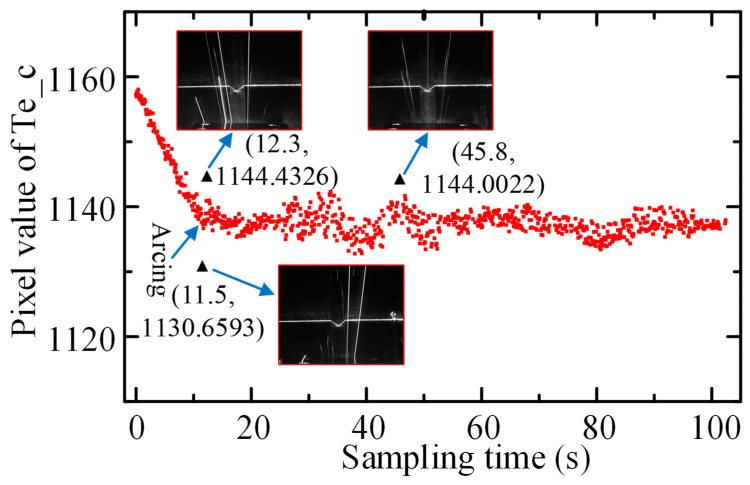
Extraction results of Te_c during tracking welding.

When tracking welding, under the action of real-time correction function, the coordinates of the seam center point in the image coordinate system *O_I_X_I_Y_I_* were theoretically constant. However, due to the response period of correction motion lagging behind the extraction period of feature points, the image coordinate value of the seam center point will fluctuate around the actual center point with varying amplitude (i.e., tracking deviation). In the actual welding process, the coordinates of the seam center point extracted from some frame images are likely to deviate abnormally from the fluctuating center, which is caused by strong arc splash interference and ROI extraction error. Figure 16 shows the real-time tracking welding results based on the above algorithm. It can be seen that in the welding process, outliers inevitably appeared, and the probability of outliers occurring at the moment of arc occurrence was much higher than that of other welding periods. The pixel coordinates of the outlier deviated from the theoretical amplitude center within 10 pixels.

Using the singular seam center point as the tracking reference point and transmitting it to the motion control system caused the welding actuator to violently vibrate and even led to safety accidents. In fact, the working environment of the optical sensor was extremely demanding, and it was difficult for us to adopt a powerful algorithm to ensure that every center point extracted by the sensor was absolutely correct. However, the position of the high-frequency image collected by the sensor was before the welding point, and the time difference brought by this “advance” made it possible to remove outliers. So an embedded Pauta criterion will be proposed in this study. The Pauta criterion was written as follows:

(17) $$\left|v_{i}\right|>2\sigma =2\sqrt{\frac{\sum_{i=1}^{n}{\left(x_{i}-\bar{x}\right)}^{2}}{n-1}}$$

where $v_{i}$ is the residual, and σ is the standard deviation.

**Figure 17:**
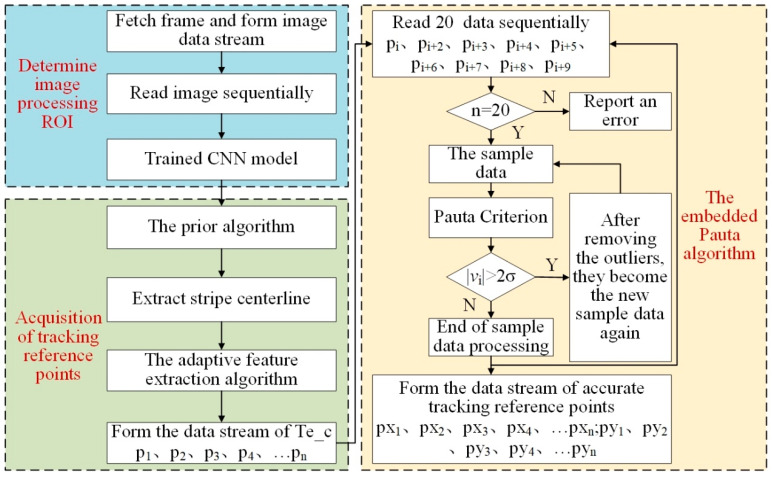
The flowchart of the embedded Pauta criterion.

As shown in Figure 17, it is the flowchart of the embedded Pauta criterion used for filtering outliers. A detailed description of the process is as follows:

(1) The data stream of Te_c was formed by the ROI determination algorithm and tracking reference point acquisition algorithm.
(2) Sequentially read 20 coordinate values from the data stream as sample data.
(3) The sample data were repeatedly processed based on the Pauta criterion (Equation (17)) until there were no outliers in the sample.
(4) Read the next sample data and repeat (2) and (3) to form the data stream of accurate tracking reference points.

**Figure 18:**
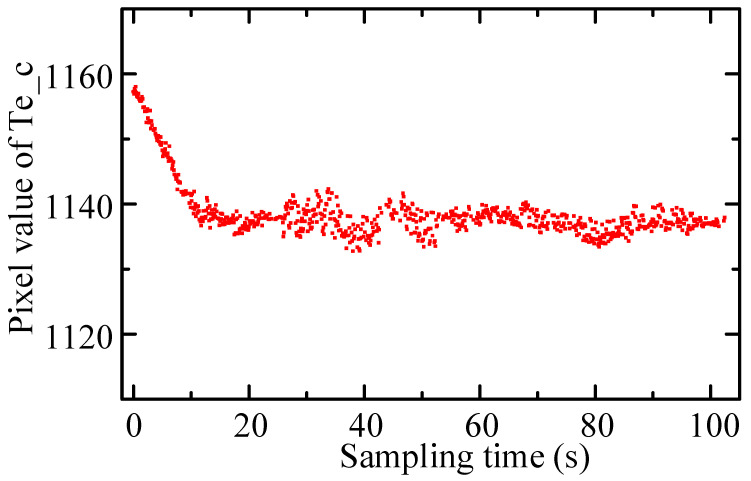
Piecewise processing results of embedded Pauta criterion.

Of 870 Te_c points from the image processing system, 41 outliers were removed (Figure 18). The Pauta criterion embedded into the real-time tracking welding system by piecewise processing eliminated the dangerous trajectory points caused by unavoidable dynamic interference and improved the robustness of tracking welding.

## 6. Experiment and Analysis

**Figure 19:**
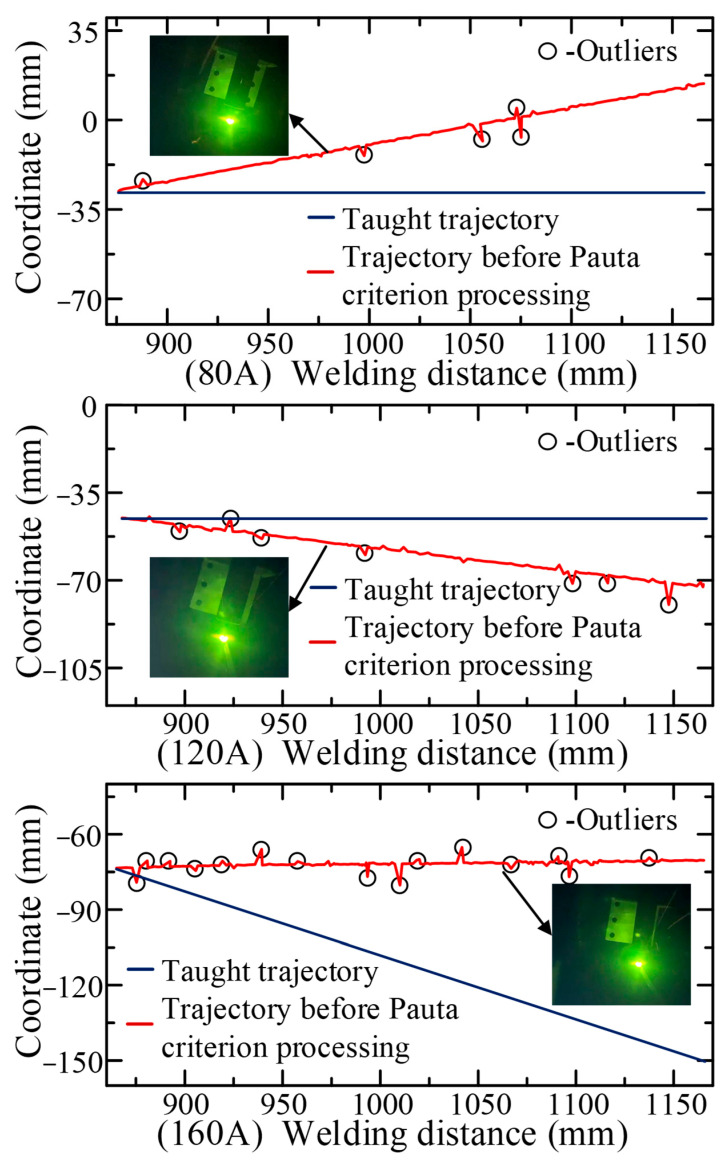
The recognition trajectory before Pauta criterion processing.

**Figure 20:**
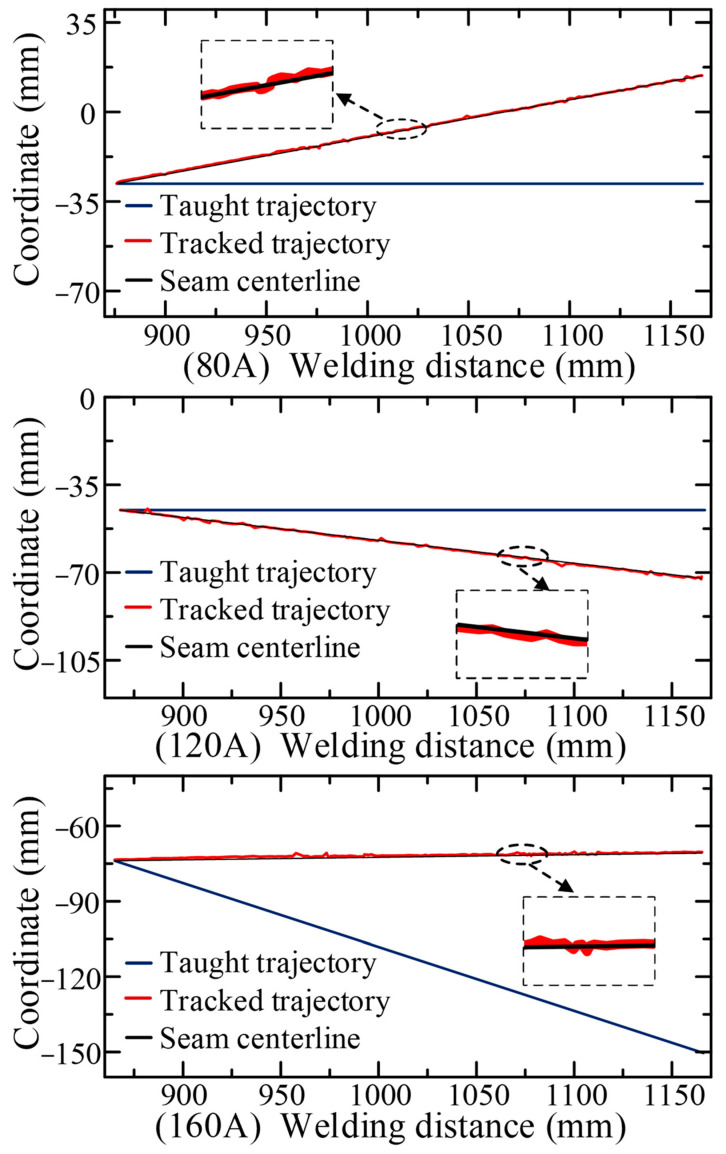
The welding trajectory under real-time tracking.

**Figure 21:**
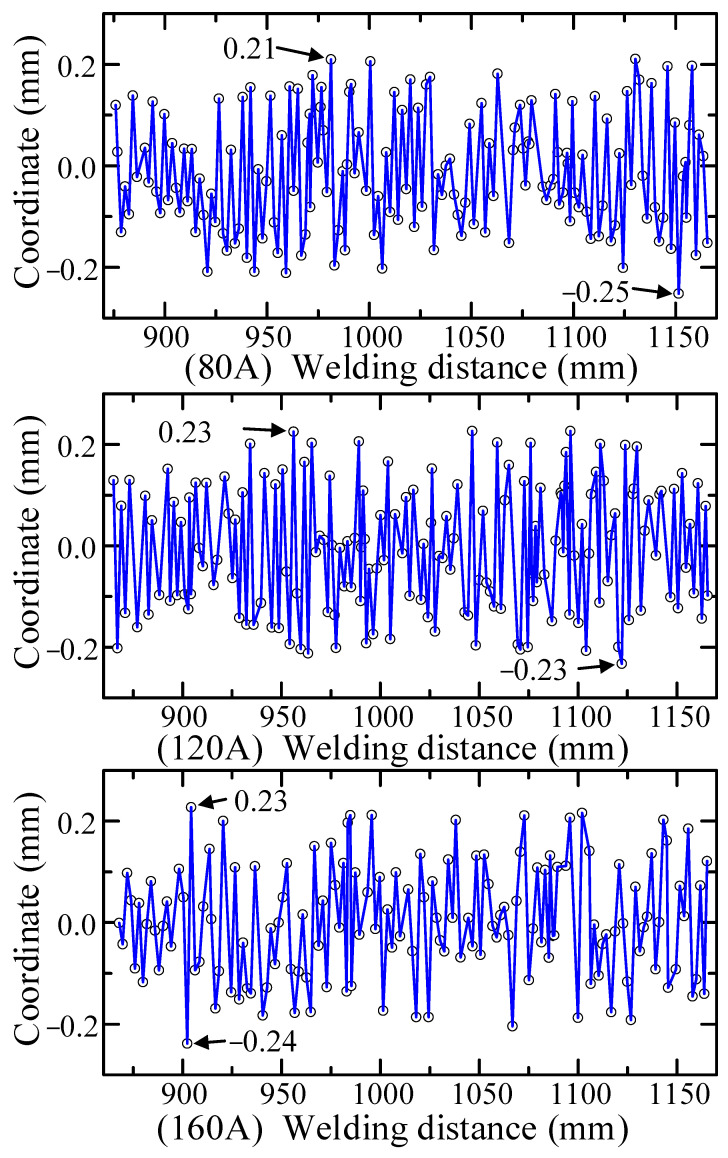
The real-time tracking accuracy.

**Figure 22:**
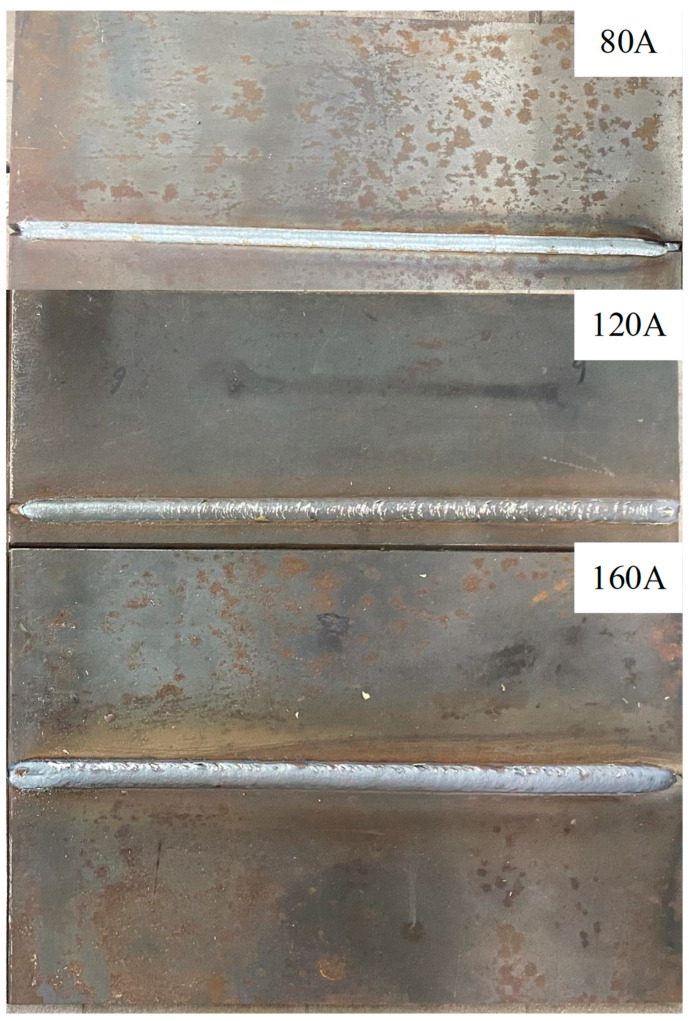
Seam tracking welding results.

In order to verify the accuracy of the CNN model, the prior model, the adaptive feature extraction algorithm, and the embedded Pauta criterion proposed in this paper, in the actual operating environment, a series of real-time tracking welding experiments based on the welding system (Figure 3) were conducted. The butt-jointed specimen with a groove angle of 60° and a plate thickness of 5 mm was used as the experimental object (Figure 5). Three groups of real-time tracking welding experiments were conducted at 80, 120 and 160 A welding current parameters. The welding speed for each group of tests was 15 cm/min, the gas flow rate was 15 L/min and the wire feed speed and welding voltage were automatically matched by the arc welder, as shown in Figure 19, Figure 20, Figure 21 and Figure 22.

The tracking welding experiment was completed under the coordinated control of the YASKAWA robot and the structured light vision sensor. From the recognition trajectory obtained by the sensor through image processing and coordinate transformation (Figure 19), it can be seen that there are 5, 7 and 15 outliers in the recognition trajectory when the welding current is 80, 120 and 160 A, respectively. The number of these outliers, which significantly deviated from the central curve of the recognition trajectory increased with the increase of welding current. If these outliers were transmitted to the tracking control system, they certainly led to the reduction of welding accuracy and even caused safety accidents.

In this experiment, the processing time of a single image was about 0.08 s (the processing period of the CNN model was about 0.03 s, the processing period of the adaptive feature extraction algorithm was about 0.04 s, and the processing period of the prior algorithm was about 0.01 s) and the processing period of the embedded Pauta criterion for 20 coordinate points was about 0.005 s. In order to be able to ease the running capacity of the tracking software, the frame rate of the camera shot could not be too high and was set to 10 fps. Since the acquisition of the real coordinate data stream preceded the execution of the robot trajectory, the communication period of the robot was slightly higher than the period of coordinate point acquisition, which was about 12 Hz.

Figure 20 shows the relationship between the welding torch tracking trajectory and the seam centerline in the tracking system with the embedded Pautu algorithm. The tracking trajectory could be obtained by recording the 3D coordinates of the robot torch endpoint at high frequency during the welding process, while the seam centerline was obtained by fitting the seam center points by least squares, which were obtained by the sensor scanning the seam before welding. When the welding current was 80, 120 and 160 A, the deviation of the endpoint of the taught trajectory from the seam was 42, 28 and 75 mm respectively. The tracked trajectory obtained by the embedded Pauta criterion had a smooth curve characteristic and no more singularities inside. Three experiments showed that the tracked trajectory was basically consistent with the seam centerline, and the algorithms mentioned above can rectify the welding torch in real time to the actual seam center.

The real-time measurement error between tracked trajectory and the seam centerline is shown in Figure 21. The measured deviation oscillated near zero with a varying amplitude throughout the welding process. The graph shows that under the same algorithmic conditions, the welding current was the key influencing parameter for singularity generation (Figure 19), but its effect on the tracking error is relatively minor. This is due to the fact that under the effect of the embedded Pauta criterion, the welding torch runs smoothly, and the deviation is maintained in a stable range without singular deviation values. The experimental results show that the maximum errors of three tracking welding experiments were −0.25, −0.23 and −0.24 mm, respectively, and the average errors were 0.1, 0.11 and 0.09 mm, respectively, which proved the overall high robustness of the proposed algorithm. The result of real-time tracking welding is shown in Figure 22. The welding position was accurate and seam metal forming was good, thereby indicating the proposed seam tracking system had outstanding performance.

## 7. Conclusions

The laser stripe, which inevitably presents different types of defect features due to intense arc and splash interference, reduced the robustness of weld tracking. Therefore, the adaptive feature point extraction algorithm and the correction algorithm were proposed in this study. Specifically, the following conclusions were drawn:

(1) Based on the principle of structured light vision, a real-time seam-tracking welding system was independently designed, which provided an important reference model for the industrial application of tracking welding technology.
(2) With a recognition rate of 97.0%, the CNN model accurately obtained the target area of image processing in real time under the different intensities of arc splash.
(3) The adaptive feature extraction algorithm based on row scanning and column scanning had strong anti-interference ability and adaptability to defects (such as holes, bulges, concaves, jumping points, etc.), welding arc and splashes in laser stripe, and accurately extracted the seam center from different types of laser stripe. The algorithm had good applicability for various types of laser stripes in real complex environments and provided a reliable solution for extracting feature points.
(4) The prior algorithm accurately and quickly located the contour of the laser stripe centerline, effectively removed other image interference and noise and improved the adaptive feature extraction algorithm to higher accuracy at the pixel level.
(5) Using the function of eliminating outliers with the embedded Pauta criterion, the structured light vision sensor, which captured high-frequency laser stripe images at the front of the welding torch, accurately obtained a smooth and gentle tracking trajectory, improved the stability of the system and avoided welding torch shaking and safety accidents. This embedded Pauta criterion provided a new idea for the safety mechanism of tracking welding, which guaranteed the stability of the system operation from the algorithmic mechanism.
(6) The accuracy verification experiment demonstrated that the tracking error was mainly controlled within ±0.2 mm, and the average error was 0.1 mm. The results confirmed that the adaptive feature extraction algorithm and the outlier removal algorithm were sufficient to ensure the accurate and reliable robot’s real-time tracking welding and provide a stable welding regime. Our future research will focus on optimizing the matching relationship between the robot control cycle and the image processing cycle to the extent that a highly robust welding application can be achieved.
